# Maternal Cardiovascular Dysfunction is Associated with Hypoxic Cerebral and Umbilical Doppler Changes

**DOI:** 10.3390/jcm9092891

**Published:** 2020-09-07

**Authors:** Giulia Masini, Jasmine Tay, Carmel M McEniery, Ian B Wilkinson, Herbert Valensise, Grazia M Tiralongo, Daniele Farsetti, Wilfried Gyselaers, Sharona Vonck, Christoph C. Lees

**Affiliations:** 1 Centre for Fetal Care, Queen Charlotte’s and Chelsea Hospital, Imperial College Healthcare NHS Trust, London W12 0HS, UK; masinigiulia@virgilio.it (G.M.); jasmine.tay@nhs.net (J.T.); 2 Fetal Medicine Unit, Careggi University Hospital, 50134 Florence, Italy; 3 Institute for Reproductive and Developmental Biology, Department of Metabolism, Digestion and Reproduction, Imperial College London, London W12 0HS, UK; 4 Division of Experimental Medicine and Immunotherapeutics, University of Cambridge, Cambridge CB2 0QQ, UK; cmm41@medschl.cam.ac.uk (C.M.M.); ibw20@medschl.cam.ac.uk (I.B.W.); 5 Department of Surgery, Division of Obstetrics and Gynecology, Policlinico Casilino, Tor Vergata, University of Rome, 00169 Rome, Italy; herbert@uniroma2.it (H.V.); grazia.tiralongo@hotmail.it (G.M.T.); danifar@hotmail.it (D.F.); 6 Department of Obstetrics and Gynecology, Ziekenhuis Oost Limburg, Schiepse Bos 6, 3600 Genk, Belgium; wilfried.gyselaers@zol.be (W.G.); sharonavonck@hotmail.com (S.V.); 7 Department of Physiology, Hasselt University, Agoralaan, 3590 Diepenbeek, Belgium; 8 Faculty of Medicine and Life Sciences, Hasselt University, Agoralaan, 3590 Diepenbeek, Belgium; 9 Department of Development and Regeneration, KU Leuven, 3000 Leuven, Belgium; 10 Centre for Fetal Care, Queen Charlotte’s and Chelsea Hospital, Du Cane Road, London W12 0HS, UK

**Keywords:** cardiovascular function, cardiac output, Doppler, fetal growth restriction, pre-eclampsia

## Abstract

We investigate the relationship between maternal cardiovascular (CV) function and fetal Doppler changes in healthy pregnancies and those with pre-eclampsia (PE), small for gestational age (SGA) or fetal growth restriction (FGR). This was a three-centre prospective study, where CV assessment was performed using inert gas rebreathing, continuous Doppler or impedance cardiography. Maternal cardiac output (CO) and peripheral vascular resistance (PVR) were analysed in relation to the uterine artery, umbilical artery (UA) and middle cerebral artery (MCA) pulsatility indices (PI, expressed as *z*-scores by gestational week) using polynomial regression analyses, and in relation to the presence of absent/reversed end diastolic (ARED) flow in the UA. We included 81 healthy controls, 47 women with PE, 65 with SGA/FGR and 40 with PE + SGA/FGR. Maternal CO was inversely related to fetal UA PI and positively related to MCA PI; the opposite was observed for PVR, which was also positively associated with increased uterine artery impedance. CO was lower (*z*-score 97, *p* = 0.02) and PVR higher (*z*-score 2.88, *p* = 0.02) with UA ARED flow. We report that maternal CV dysfunction is associated with fetal vascular changes, namely raised impedance in the fetal-placental circulation and low impedance in the fetal cerebral vessels. These findings are most evident with critical UA Doppler changes and represent a potential mechanism for therapeutic intervention.

## 1. Introduction

Maternal cardiovascular function changes profoundly throughout pregnancy [1]. Cardiac output typically increases until the early third trimester, while the opposite is observed for peripheral vascular resistance [2]. In healthy women who develop fetal growth restriction (FGR), cardiac output is lower than expected, and peripheral vascular resistance is increased. When pre-eclampsia occurs without FGR, high cardiac output and low peripheral vascular resistance [3] are described. These maternal cardiovascular changes are associated with maternal and fetal Doppler changes; higher uterine artery and umbilical artery Doppler impedance is related to increased maternal peripheral vascular resistance and reduced maternal cardiac output [4]. These results from our group were obtained from one carefully phenotyped cohort of women recruited in a single centre by measuring maternal cardiovascular parameters using an inert gas rebreathing technique in the standing maternal position.

Several non-invasive techniques are available for the measurement of cardiovascular parameters in pregnant women. There currently is no gold standard: the choice depends on factors such as the availability of techniques, training of the operators and costs. Some techniques can be used only in the maternal lying position [5], others in the standing position or during exercise [6]; others allow continuous monitoring [7]. Furthermore, the definitions of FGR and pre-eclampsia vary from study to study. Within this heterogeneous milieu of techniques and definitions, the results of studies on maternal haemodynamic changes using different methodologies are not easily generalizable [8,9], and true relationships may be obscured.

In this study, we analysed cardiac output and peripheral vascular resistance in a cohort of pregnant women with or without pre-eclampsia or FGR. We used three different techniques for cardiovascular assessment in pregnancy, based on inert gas rebreathing [10,11], continuous Doppler [12,13] and impedance cardiography [14]. These techniques cannot be used interchangeably [9], so we have adjusted the maternal cardiovascular results in relation to the gestational week, based on technique-specific healthy controls. Our hypothesis is that increased maternal peripheral vascular resistance and reduced maternal cardiac output are associated with Doppler changes in the fetal circulation, denoting progressively worsening hypoxia. Our aim was to investigate the relationship between maternal cardiovascular function and fetal-placental Doppler indices in women with healthy or pathological pregnancy outcome and where fetuses showed extreme Doppler changes, namely absent or reversed end diastolic (ARED) flow in the umbilical artery.

## 2. Materials and Methods

This is a prospective cohort observational study performed in three European University Hospitals, located in London, UK (Centre A), Hasselt, Belgium (Centre B) and Rome, Italy (Centre C), and included women with singleton pregnancies at or above 24 weeks’ gestation, who were healthy or affected by one of the following conditions: pre-eclampsia alone; small for gestational age (SGA) fetuses/FGR alone; or the combination of both pre-eclampsia and SGA/FGR.

Before the combined analysis of the datasets, the study analysis plan was to examine maternal cardiac output and peripheral vascular resistance in relation to maternal (uterine) and fetal (umbilical and middle cerebral artery) Doppler impedance indices.

The data for the women in Centre A have been recently reported [4], though analyses combining data for all three centres have not been undertaken before.

Women were recruited from January 2013 and the studies were individually approved by each local Research Ethics Committee (Centre A: National Research Ethics Service Committee London Riverside, ref 15/LO/0341, 17/04/2015; Centre B: MEC ZOL, ref 13/090 U, 16/09/2013; Centre C: Ethic Committee Lazio 2, ref 82.17, 13/06/2017). Exclusion criteria were the presence of multiple pregnancy, fetal malformations and maternal cardiovascular comorbidities, such as smoking or chronic hypertension.

Pre-eclampsia was defined as a maternal blood pressure at diagnosis of >140/90 mm Hg and a urine protein creatinine ratio of >30; SGA was defined as abdominal circumference or estimated fetal weight <10th centile with normal umbilical artery Doppler. The presence of abnormal umbilical artery Doppler (above the 95th percentile) in an SGA fetus denoted the presence of FGR [15,16]. Pre-eclampsia, SGA and FGR were considered to be pathological pregnancy outcomes.

All women underwent a fetal ultrasound scan and cardiovascular assessment within 72 h of the scan, as detailed below.

### 2.1. Ultrasound Assessment

Fetal ultrasound scans were performed using Samsung WS80 (Samsung Medison, Seoul, Republic of Korea) in Centre A, General Electric Voluson E8 Expert (GE Healthcare, Machelen, Belgium) in Centre B and General Electric Voluson E6 (GE Healthcare, Milan, Italy) in Centre C. Measurements of fetal biparietal diameter, head circumference, abdominal circumference and femur length were obtained, and fetal growth was assessed using local growth charts.

With regards to Doppler assessment, uterine artery mean pulsatility index (PI), umbilical artery PI and middle cerebral artery (MCA) PI were collected. Doppler parameters were analysed in all groups [17]; umbilical and MCA PI values were transformed into the correspondent *z*-score by gestational week as previously described [4]. Uterine artery mean PI results were analysed untransformed, as there is no significant change with gestational week after 24 weeks’ gestation.

### 2.2. Cardiovascular Assessment

Cardiac output and peripheral vascular resistance were measured with different devices according to the technique available at each centre after 5 to 10 min of rest, with the participant in the left lateral lying position to avoid aortocaval compression. Standing measurements were made where this was feasible, depending on the device used, again after a period of rest for stabilization of cardiovascular function.

Centre A: an operator-independent device based on inert gas rebreathing (Innocor; Innovision A/S, Glamsbjerg, Denmark) was used for the measurement of cardiac output as previously described [1,18]. This device allows measurements to be taken both in lying and standing positions. After the measurement of brachial blood pressure with a separate device (Omron M-7; OMRON Healtcare Europe BV, Hoofddorp, The Netherlands), peripheral vascular resistance was derived with the formula:

Peripheral vascular resistance = (mean arterial pressure × 80)/cardiac output

Centre B: an automated and operator-independent device measured both blood pressure through oscillometry and cardiac output through impedance cardiography (Non-Invasive Continuous Cardiac Output Monitor, NICCOMO, Medis Medizinische Messtechnik GmbH, Ilmenau, Germany). Inter- and intra-observer variation as well as normal reference values have been reported elsewhere [19,20,21]. Blood pressure was measured on the right arm with an appropriate cuff width at standard time points. For the measurement of cardiac output, four electrodes (two on the axillary line under the thorax and two in the neck) were used to transmit an alternating current with very low amplitude and high frequency through the maternal thorax, eliminating skin resistance. The voltage produced by this passage was recorded as an electrocardiogram and an impedance cardiogram. Heart rate was calculated from the electrocardiogram, stroke volume from an internal algorithm based on the impedance cardiogram [22] and cardiac output from the formula:

Cardiac output = heart rate × stroke volume

Peripheral vascular resistance was calculated from mean arterial pressure and cardiac output as per the formula above. Both lying and standing measurements were obtained with this device.

Centre C: cardiac output was measured with a continuous Doppler-based device (UltraSonic Cardiac Output Monitor, USCOM, USCOM Ltd., Coffs Harbour, Australia), which is non-invasive, though not operator-independent. A non-imaging continuous-wave Doppler transducer was placed at the suprasternal notch to measure transaortic blood flow. After manual entry of the patient’s weight and height, the device computed cardiac output from the velocity time integral of the blood flow through the aortic valve [23]. Blood pressure was measured separately (Logiko Digit Device, Moretti S.p.A., Cavriglia, Italy) and then added to the algorithm for the automatic calculation of peripheral vascular resistance. This device allows measurement of cardiovascular parameters in a lying position only.

In order to compare the measurements of cardiac output and peripheral vascular resistance obtained with the three different techniques, for each subject the absolute values of these parameters were transformed into the corresponding *z*-score for gestational week. For each week, the mean and standard deviation used as references for *z*-scoring were calculated from those obtained in a group of women with healthy pregnancies assessed in the same centre and using the same methodology [3,4].

### 2.3. Statistical Analysis

Statistical analysis was performed with SPSS statistical software (Version 25 SPSS Inc, Chicago, IL, USA). Continuous variables were described as mean (standard deviation) or median (range) where appropriate. Demographic characteristics and maternal cardiovascular indices between controls and pathology groups were compared with the Kruskal–Wallis test. A further analysis was performed for comparison of cardiovascular measurements between those cases with positive umbilical artery end diastolic flow (EDF) and those with ARED; *t*-test was used to compare means.

Associations between maternal cardiovascular parameters and Doppler indices were assessed using polynomial regression analyses. By using fitted curves, we chose quadratic models to describe these relationships. A *p*-value of less than 0.05 was considered statistically significant; in order to control the Type I error, we used the Bonferroni correction in the case of multiple comparisons.

For all analyses, we removed 3 cases with implausible outlying maternal cardiovascular values: one case with a cardiac output *z*-score of 10.6, and two cases with peripheral vascular resistance *z*-scores of 10.99 and 34.77.

## 3. Results

**Figure 1:**
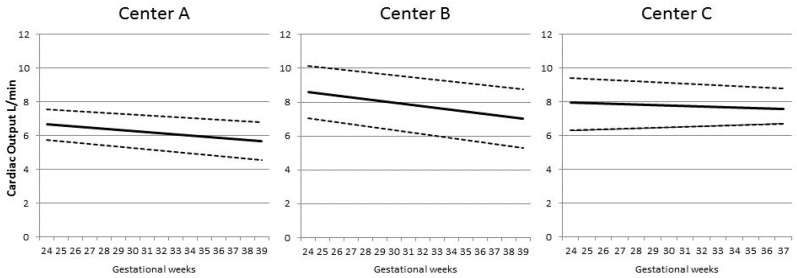
The figure shows the trend of maternal cardiac output in relation to gestational week for Centre A, B and C, respectively. Differences in trends represent the different proportion of controls and cases with preeclampsia, small for gestational age/fetal growth restriction or both in the three centres. Mean ± 1 SD (L/min).

**Figure 2:**
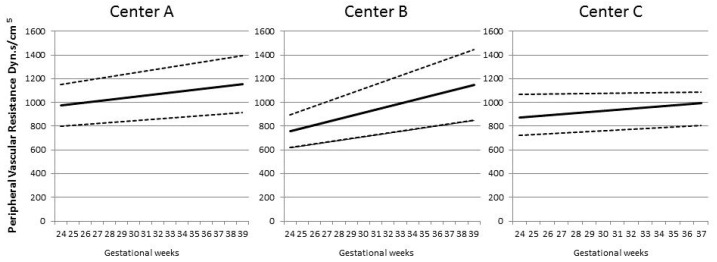
The figure shows the trend of maternal peripheral vascular resistance in relation to gestational week for Centre A, B and C, respectively. Differences in trends represent the different proportion of controls and cases with preeclampsia, small for gestational age/fetal growth restriction or both in the three Centres. Mean ± 1 SD (dyn.s/cm^5^).

A total of 233 participants were included, 107 in Centre A, 98 in Centre B and 28 in Centre C. Of those, 81 were women with control (healthy) pregnancies and 152 had pathological outcomes (47 with pre-eclampsia, 65 with SGA/FGR and 40 with pre-eclampsia and SGA/FGR). The trends with gestational age for CO and PVR are shown in Figure 1 and Figure 2.

No differences were found between the pathological pregnancy outcome groups with regards to maternal age, body mass index and gestational age at cardiovascular and scan investigations (Table 1).

Gestational age at delivery and birthweight in women with pathological pregnancy outcomes were significantly lower than for the controls (*p* < 0.001 for all groups vs. controls). Although not significant, median gestational age at cardiovascular and scan assessment, gestational age at delivery and birthweight were lower in the pre-eclampsia with SGA/FGR group.

### 3.1. Cardiovascular Changes in Pathological Pregnancy Outcome

A comparison of cardiovascular parameters is detailed in Table 2.

A total of 231 lying and 205 standing cardiac output *z*-score values were compared. No differences in lying cardiac output were found between controls and women with pathological outcomes. Standing cardiac output was significantly lower in pre-eclampsia + SGA/FGR compared to controls (−0.82 ± 1.24, *p* = 0.002).

For peripheral vascular resistance, 229 lying and 205 standing *z*-score values were analysed. Women with pathological outcomes showed higher lying peripheral vascular resistance than controls. Standing vascular resistance *z*-scores were also significantly higher in pre-eclampsia + SGA/FGR compared to controls and SGA/FGR (2.96 ± 3.27, *p* < 0.001).

**Figure 3:**
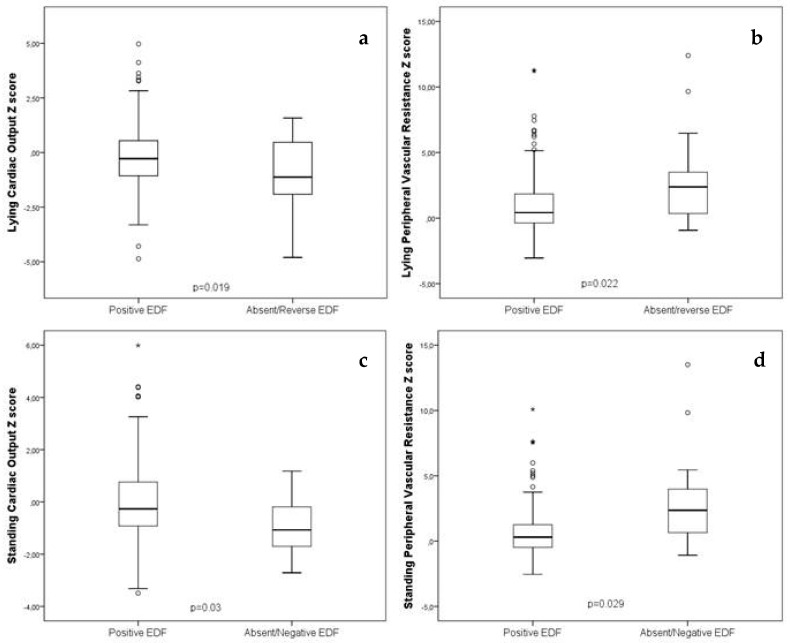
(From left to right): positive end diastolic flow (EDF) vs. absent or reversed EDF in the umbilical artery: (**a**) lying cardiac output *z*-score, (**b**) lying peripheral vascular resistance *z*-score, (**c**) standing cardiac output *z*-score, (**d**) standing peripheral vascular resistance *z*-score.

Cardiac output and peripheral vascular resistance *z*-scores were compared in the whole study population, divided into two subgroups characterized by a different EDF pattern in the umbilical artery. Cardiac output was lower and vascular resistance higher in the ARED group, both in lying and standing positions (Table 3, Figure 3).

### 3.2. Association between Maternal Cardiovascular Parameters and Doppler Indices

Umbilical artery Doppler PI was available in 228 subjects, middle cerebral artery PI in 137 subjects, of whom 21 were controls, and uterine artery mean PI in 196 subjects, of whom 68 were controls. For this analysis, all measurements were analysed in aggregate, without distinction in terms of pregnancy outcome.

The association between fetal and maternal Doppler indices and cardiac output are reported in Table 4.

Umbilical artery PI *z*-score was inversely associated with maternal standing cardiac output *z*-score (*r*^2^ = 0.036, *p* = 0.026). Middle cerebral artery PI *z*-score was positively associated with lying cardiac output *z*-score (*r*^2^ = 0.052, *p* = 0.029). No associations were detected between uterine artery mean PI and cardiac output.

The relationship between umbilical, middle cerebral and uterine artery Doppler PI and peripheral vascular resistance are shown in Table 5.

Uterine artery PI showed a significant and non-linear association with both lying and standing peripheral vascular resistance *z*-scores (*r*^2^ 0.049 with *p* = 0.009 and *r*^2^ 0.054 with *p* = 0.011, respectively). The same association was noted for umbilical artery PI *z*-score (*r*^2^ 0.06, *p* = 0.001 compared with lying maternal peripheral vascular resistance *z*-score and *r*^2^ 0.082, *p* < 0.001 compared with standing *z*-score). Middle cerebral artery PI *z*-score was inversely associated with changes in lying peripheral vascular resistance *z*-score (*r*^2^ 0.062, *p* = 0.016).

## 4. Discussion

In this exploratory study, fetal-placental Doppler indices are associated with maternal cardiac output and vascular resistance both in healthy pregnancies and those with pathological outcomes. The importance of this study is that it utilizes data from three distinct populations and three different techniques for generalizability. Previously unreported is that an abnormal maternal cardiovascular function is associated with reduced impedance in fetal cerebral vessels. Cerebral vasodilatation is the fetal compensatory response to hypoxia detected by direct oxygen sensing of the cerebral vasculature, not mediated through the autonomic system. It is therefore plausible that cerebral redistribution is associated with restricted uteroplacental perfusion secondary to impaired maternal cardiovascular function, hence manifesting as fetal hypoxia. Though the absolute magnitude of the associations seen is small, in the context of fetal growth restriction and pre-eclampsia, marginal changes in the utero-placental circulation may lead to major perturbations in the fetal circulation.

The most severe changes in maternal cardiovascular parameters are observed when ARED flow is seen in the fetal umbilical circulation. This is commonly seen in women with the dual pathology of both pre-eclampsia and SGA/FGR, where maternal cardiac output is low and vascular resistance is high. Analysis of cardiovascular parameters in women with or without positive EDF in the umbilical artery revealed that maternal cardiac output was significantly lower where the fetal umbilical artery blood flow was severely compromised showing ARED flow, and these women also had the highest vascular resistance. These Doppler changes in pre-eclampsia, particularly with FGR, have hitherto been ascribed to placental damage and abnormal development [24]. Villous architecture in FGR is abnormal with poorly branched villi [25]. However, histopathological changes in the placenta in these pathological outcome pregnancies are not reproducibly reported, nor are they consistent [26]. Though it cannot be denied that the placenta, as the organ of gaseous exchange, must play a part in the process, there are few data to quantify the extent of this. Our findings build on those of previous studies [4,27] indicating that rather than the placenta, maternal cardiovascular function modulates uterine artery and fetal vascular function, being associated with impaired utero-placental perfusion and, consequent upon this, fetal hypoxia/acidaemia.

We assessed cardiac output and peripheral vascular resistance in three centres with different methodologies. It is likely that the magnitude of the findings that we report is attenuated as the absolute CO and PVR values were different in the three centres. However, the trends with gestational age were similar (Figure 1 and Figure 2), thus are likely to be generalizable to centres using different techniques. The use of these techniques also allowed the assessment of the same maternal cardiovascular parameters in two different positions, lying with all devices and standing with two, namely inert gas rebreathing and impedance cardiography. Posture changes influence cardiovascular response and can differentiate between normal and hypertensive pregnancies. Healthy pregnancies are characterized by an increase in cardiac output when standing. By contrast, the orthostatic changes are less marked in early pre-eclampsia [14]. We confirm that the more severe forms of pre-eclampsia, being those associated with FGR, are associated with a paradoxical decrease in CO in standing position. The putative explanation for this observation is that cardiac contractility is altered in pre-eclampsia compared with normal pregnancies, as studies based on conventional or speckle tracking echocardiography have suggested [28]. We also observed an increase in PVR in all pathological outcome categories, probably as a result of reduced arterial compliance [29].

Our findings provide an explanation for previously reported observations. Beta blockers, which have negative chronotropic effects on maternal cardiac output, are associated with FGR [30] and stillbirth. Vasodilatation and intravascular volume expansion in conditions where there is increased vascular resistance may increase fetal growth and prolong pregnancy [31]. If maternal cardiovascular function does indeed modulate uteroplacental perfusion in conditions characterized by abnormal fetal oxygen and acid/base status Doppler findings, then therapeutic intervention could allow an approach to fetal therapy in conditions previously thought to be due to irreversible placental disease.

## 5. Conclusions

Lower maternal cardiac output and higher peripheral vascular resistance are associated with the fetal circulatory changes that occur in response to hypoxia, namely an increased impedance in the umbilical vessels and reduced impedance in the cerebral arteries. This becomes particularly obvious when umbilical Doppler findings are critically abnormal. This relationship raises the potential for therapeutic manipulation of maternal cardiovascular function in order to improve pathologically abnormal uteroplacental function, and hence fetal condition.

## Figures and Tables

**Table 1:** Demographic characteristics of study participants.

Characteristic	Controls	Pre-Eclampsia	SGA/FGR	Pre-Eclampsia + SGA/FGR	Kruskal–Wallis *p*-Value
*N*.	81	47	65	40	-
Mean maternal age, years (SD)	32.9 (4.5)	31.6 (4.4)	32.1 (5.2)	31.9 (5.1)	0.276
Median BMI (range)	23.9 (21.2–25.9)	25.2 (22.7–29.1)	24.1 (21.5–28.7)	23.56 (22.5–27)	0.082
N. of nulliparous (%)	38 (46.9)	39 (83)	38 (58.5)	24 (60)	-
Median gestational age at CV test, weeks (IQR)	33.6 (29.3–36.6)	32.7 (28.1–35.9)	32 (30.3–34.1)	30.7 (26.6–33.5)	0.026
Median gestation at delivery, weeks (IQR)	39 (38–40)	34.3 (31.6–37) ^1^	36.6 (32.6–37.9) ^1^	33.1 (27.7–35.8) ^1^	<0.001
Median birthweight, g (IQR)	3320 (3038.8–3552.5)	1877.5 (1339.8–2685) ^1^	1990 (1280.5–2307.5) ^1^	1333 (704–1910) ^1^	<0.001

SGA, small for gestational age; FGR, fetal growth restriction; SD, standard deviation; IQR, interquartile range; CV, cardiovascular. ^1^ *p* < 0.001 compared to controls.

**Table 2:** Comparison of cardiac output and peripheral vascular resistance *z*-scores in pathological outcome groups.

Variable	Controls		Pre-Eclampsia		SGA/FGR		Pre-Eclampsia + SGA/FGR		Kruskal–Wallis *p*-Value
	*N*.	Mean *z*-Score (SD)	*N*.	Mean *z*-Score (SD)	*N*.	Mean *z*-Score (SD)	*N*.	Mean *z*-Score (SD)	
CO lying	81	−0.067 (0.96)	45	−0.009 (1.74)	65	−0.68 (1.34)	40	−0.51 (1.69)	0.007
CO standing	81	0.02 (1.04)	47	0.35 (2.006)	44	−0.48 (1.24)	33	−0.82 (1.24) ^1^	0.002
PVR lying	81	0.087 (0.92)	45	1.696 (2.6) ^2^	64	1.52 (2.48) ^2^	39	2.56 (2.72) ^2^	<0.001
PVR standing	81	0.037 (0.92)	47	1.22 (2.26)	44	0.83 (1.84)	33	2.96 (3.27) ^3^	<0.001

SGA, small for gestational age; FGR, fetal growth restriction; SD, standard deviation; CO, cardiac output; PVR, peripheral vascular resistance. ^1^ *p* < 0.0125 compared to controls. ^2^ *p* < 0.001 compared to controls. ^3^ *p* < 0.001 compared to controls and SGA/FGR.

**Table 3:** Comparison of cardiac output and peripheral vascular resistance *z*-score in cases with positive end diastolic flow and cases with absent/reverse end diastolic flow in the umbilical artery.

Variable	Positive EDF		Absent/Negative EDF		*p*-Value (*T*-Test)
	*N*.	Mean (SD)	*N*.	Mean (SD)	
Lying CO	192	−0.22 (1.41)	22	−0.97 (1.57)	0.019
Lying PVR	191	1.02 (2.12)	21	2.88 (3.39)	0.022
Standing CO	174	−0.03 (1.49)	14	−0.91 (1.04)	0.03
Standing PVR	174	0.69 (1.89)	14	3.33 (4.01)	0.029

EDF, end diastolic flow; SD, standard deviation; CO, cardiac output; PVR, peripheral vascular resistance.

**Table 4:** Association between cardiac output and Doppler pulsatility indices.

Doppler Variable	Cardiovascular Variable	*r* ^2^	*p*-Value	Regression Equation
Uterine artery PI	Lying CO *z*-score	0.007	0.499	y = −0.005x + 0.008x^2^ + 0.894
	Standing CO *z*-score	0.024	0.135	y = −0.037x + 0.015x^2^ + 0.868
Umbilical artery PI	Lying CO *z*-score	0.008	0.407	y = −0.14x + 0.013x^2^ + 1.466
	Standing CO *z*-score	0.036	0.026	y = −0.343x + 0.027x^2^ + 1.280
Middle cerebral artery PI	Lying CO *z*-score	0.052	0.029	y = 0.106x − 0.056x^2^ − 0.669
	Standing CO *z*-score	0.018	0.389	y = 0.135x − 0.016x^2^ − 0.826

PI, pulsatility index; CO, cardiac output.

**Table 5:** Association between peripheral vascular resistance and Doppler pulsatility indices.

Doppler Variable	Cardiovascular Variable	*r* ^2^	*p*-Value	Regression Equation
Uterine artery PI	Lying PVR *z*-score	0.049	0.009	y = 0.031x + 0.001x^2^ + 0.870
	Standing PVR *z*-score	0.054	0.011	y = 0.060x − 0.004x^2^ + 0.867
Umbilical artery PI	Lying PVR *z*-score	0.06	0.001	y = 0.310x − 0.01x^2^ + 1.234
	Standing PVR *z*-score	0.082	<0.001	y = 0.433x − 0.02x^2^ + 1.102
Middle cerebral artery PI	Lying PVR *z*-score	0.062	0.016	y = −0.156x + 0.003x^2^ − 0.613
	Standing PVR *z*-score	0.045	0.088	y = −0.167x + 0.007x^2^ − 0.722

PI, pulsatility index; PVR, peripheral vascular resistance.
